# Placenta-Derived Extracellular Vesicles (pdEVs): Key Mediators That Affect the Metabolic Health of Offspring in Early Nutritional Environments

**DOI:** 10.3390/biom16060826

**Published:** 2026-06-02

**Authors:** Hanmo Lin, Chuhan Shao, Jie Yu, Haiyan Chen, Yaolin Ren, Jing Ren, Yuan Zeng, Yifan Wu, Qian Zhang, Xinhua Xiao

**Affiliations:** Key Laboratory of Endocrinology, Ministry of Health, Department of Endocrinology, Peking Union Medical College Hospital, Peking Union Medical College, Chinese Academy of Medical Sciences, Beijing 100730, China; s2025001030@student.pumc.edu.cn (H.L.); sch999@student.pumc.edu.cn (C.S.); b2023001044@student.pumc.edu.cn (J.Y.); s2024001029@student.pumc.edu.cn (H.C.); s2023001083@pumc.edu.cn (Y.R.); renjing@student.pumc.edu.cn (J.R.); b2024001052@student.pumc.edu.cn (Y.Z.); pumc_wuyifan@student.pumc.edu.cn (Y.W.)

**Keywords:** placenta, extracellular vesicles, exosome, metabolic programming, offspring, maternal nutrition

## Abstract

Placenta-derived extracellular vesicles (EVs), particularly exosomes, serve as key mediators that influence metabolic programming in offspring under adverse early nutritional conditions, such as maternal obesity or gestational diabetes. They respond to maternal nutritional disturbances—such as obesity or gestational diabetes—by altering the composition of the miRNAs and proteins they carry. Evidence from in vivo and in vitro studies suggests that these modified EVs influence offspring metabolic programming through multiple putative pathways: regulating fetal pancreatic β-cell development and function, modulating lipogenesis via PPARγ signaling, affecting placental angiogenesis, and promoting inflammation and epigenetic alterations. By transmitting maternal environmental signals to the fetus, placental EVs are hypothesized to contribute to long-term metabolic phenotypes and disease susceptibility. This review critically examines the current evidence positioning placental EVs as key messengers in maternal–fetal communication, evaluates the strength of evidence supporting their role in shaping offspring metabolic health, identifies major knowledge gaps (e.g., limited direct evidence in human offspring, lack of standardized isolation methods), and suggests their potential as early intervention biomarkers or therapeutic targets for preventing metabolic disorders in offspring. We also highlight the need for prospective cohort studies and mechanistic validation in appropriate animal models to establish causality.

## 1. Introduction

In 1989, Professor Barker summarized the association between fetal growth and the susceptibility to cardiovascular diseases and proposed the well-known Developmental Origins of Health and Disease (DOHaD) hypothesis, also known as developmental programming [1,2]. This hypothesis posits that adverse environmental exposures during critical periods of early development—particularly in utero—are associated with long-term alterations in physiology, metabolism, and disease risk [3,4]. The DOHaD hypothesis offers a novel perspective on the pathogenesis of chronic diseases, advancing the disease spectrum and drawing attention to the impact of the early stage of life on metabolic diseases. Currently, researchers have identified multiple exposure factors associated with disease risk, especially early in the nutritional environment [5,6].

Early nutritional conditions—including maternal obesity, diabetes, a high-fat diet, and malnutrition—are significantly related to the risk of metabolic conditions such as offspring obesity, insulin resistance, and type 2 diabetes mellitus (T2DM). The well-known Dutch famine studies provided landmark evidence: individuals exposed to prenatal starvation exhibited increased blood glucose levels and a higher risk of diabetes in adulthood [7]. Similarly, cohort studies conducted among women with obesity or gestational diabetes mellitus (GDM) indicate that children born to these mothers have elevated rates of obesity or early onset diabetes [8]. Research is also ongoing into other nutritional factors (e.g., trace elements), and their intergenerational mechanisms, with current evidence pointing toward signal transduction and epigenetic modifications as key factors [9].

The placenta is the only interface connecting the mother and fetus, with dual origins from both maternal and fetal tissues [10]. Traditionally, it serves as the whole conduit for maternal–fetal material exchange and nutrient transport and performs endocrine [11], metabolic [12], and immune [13] functions. Furthermore, the placenta is recognized as a programming agent for the fetal phenotype in response to alternative environments. Therefore, the specific signaling pathways through which environmental changes induce metabolic alterations represent a key focus of future research, which is crucial for elucidating the mechanisms of metabolic programming and identifying viable therapeutic targets.

In recent years, cellular signal transduction mediated by extracellular vesicles (EVs)—a heterogeneous population of membrane-bound particles released by cells—has emerged as a focus of research. EVs are broadly classified into three major categories based on their biogenesis: exosomes (40–160 nm, originating from the endocytic pathway), microvesicles (50–1000 nm, shed from the plasma membrane), and apoptotic bodies (50–5000 nm, released during programmed cell death) [14,15]. Among these, exosomes have been the most extensively characterized in the context of pregnancy. Exosomes can carry various cargoes to facilitate intercellular communication in adjacent tissues or enter the circulation to influence distant cells, which help maintain homeostasis [16].

During gestation, placenta-derived EVs (pdEVs) represent a significant source of maternal circulating EVs and serve as key signaling intermediates in maternal–fetal communication [17]. An increasing amount of research has focused on the effects of pdEVs in adverse maternal conditions such as GDM, preeclampsia, fetal growth restriction, and preterm birth. These findings collectively suggest that pdEVs may function as critical signaling molecules involved in metabolic programming. However, it is important to note that most existing evidence derives from maternal circulation studies or in vitro experiments; direct evidence linking pdEVs to offspring metabolic outcomes in humans remains limited. Furthermore, methodological heterogeneity in EV isolation and characterization (e.g., ultracentrifugation vs. size-exclusion chromatography) poses challenges for cross-study comparability.

This review focuses on pdEV-mediated cellular communication under different early nutritional environments, with particular emphasis on their putative mechanisms of action in fetal metabolic programming. We critically synthesize current evidence, identify major knowledge gaps, and discuss potential directions for the early prevention of and future intervention for metabolic disorders.

## 2. Placenta—The Only Interface Connecting the Mother and Fetus

### 2.1. Structure and Function of the Placenta

The placenta is a transient support organ that is formed during pregnancy and is jointly formed by both the mother and the fetus. It is composed of the chorion, which developed from the blastocyst (fetus origin), and the basal decidua of the maternal endometrium (maternal contribution). Placental formation and development is a continuous process that occurs simultaneously with embryonic development [18,19,20]. It is a highly multifunctional organ that takes up the nutrients required by the fetus, regulates the exchange of respiratory gases, removes carbon dioxide and other metabolic waste from the fetus, and protects the fetus against maternal immunity [13,21,22]. It also performs endocrine [11] and metabolic [12] functions, secreting multiple hormones [23,24]—primarily human chorionic gonadotropin (hCG), human placental lactogen (hPL), and placental growth hormone (hPGH)—to maintain pregnancy and regulate fetal growth and development while exerting feedback effects on the mother that alter her metabolic state. Beyond its role in nutrient and hormone transport, the placenta serves as an immunological barrier at the maternal–fetal interface.

With a more in-depth understanding of placental biology, it is now widely recognized that the placenta is not merely a simple conduit and temporary organ but rather the primary “sensor” and “processor” of the early nutritional environment [25]. It dynamically adjusts its structure and metabolic state in response to changes in the external environment [26]. These functional adaptations are hypothesized to play crucial roles in the metabolic programming of the offspring.

### 2.2. Changes in the Adaptability of the Placenta to Early Nutritional Condition

The placenta brings the maternal and fetal blood streams into proximity with only a few cell layers in the thinnest area, which makes it highly sensitive to disturbances in the external environment and results in corresponding adaptive changes when environmental conditions are altered to ensure the survival of the fetus. This adaptation is particularly pronounced when individuals suffer from intrauterine nutritional restriction. The primary changes involve morphological alterations, such as modifications in placental weight and vascular density. Research has indicated that in an insufficient nutritional environment, placental weight increases, probably increasing the energy supply to sustain fetal growth [27]. The transport rate subsequently adjusts to accommodate nutritional demands in response to shifting nutrient availability. Glucose is transferred across the placental barrier by facilitated diffusion; thus, glucose flux to the fetus depends mainly on the concentration gradient across the placenta as well as the density of glucose transporters (GLUTs) in the fetal basal plasma membrane. In cases of maternal malnutrition during gestation, GLUT1 protein levels increase to optimize the placental glucose supply [27]. In addition, the placenta releases hormones into the maternal blood to increase maternal blood pressure, forcing more nutrients across the placental surface [28]. Moreover, in pregnancies complicated by GDM, distinct and opposing adaptations are observed. Maternal hyperglycemia may lead to fetal hyperinsulinism, which increases fetal oxygen demand. In response, the placental surface area increases to ensure adequate oxygen transfer. Simultaneously, GLUT1 expression and activity are upregulated to adapt to maternal hyperglycemia, which may paradoxically stimulate excessive fetal growth [29,30]. The mechanisms underlying these adaptations are not fully understood but are thought to involve DNA methylation and endocrine modulation. Notably, similar adaptive changes have also been reported in pregnancies complicated by preeclampsia (PE) and fetal growth restriction (FGR), although with distinct molecular signatures [31].

### 2.3. Placenta-Derived Extracellular Vesicles (pdEVs): An Emerging Maternal–Fetal Signaling Molecule

Placenta-derived extracellular vesicles (pdEVs) refer to the population of EVs originating from placental tissues that enter maternal circulation during pregnancy. These vesicles include exosomes, and apoptotic bodies, with exosomes being the most extensively characterized subtype in the context of maternal–fetal communication.

#### 2.3.1. Biogenesis, Classification, and Characterization of Extracellular Vesicles

Extracellular vesicles (EVs) constitute a heterogeneous population of nanometer-sized membrane vesicles that are actively released by cells and are involved in various biological processes [32]. EVs encapsulate cargo—including lipids, proteins, RNA, DNA, bioactive enzymes, and small molecules—within a phospholipid bilayer and transport them to recipient cells [14]. Consequently, EVs are considered important mediators of intercellular communication [33] and potential biomarkers for disease diagnosis and prognosis [34]. On the basis of their biogenesis, size and release process, EVs can be divided into three major categories: exosomes (40~160 nm in diameter), which originate from the endocytic pathway; microvesicles (MVs), also known as ectosomes (50~1000 nm in diameter), which are derived from the plasma membrane; and apoptotic bodies (50~5000 nm in diameter), which are released by cells undergoing apoptosis [14,15].

**Figure 1:**
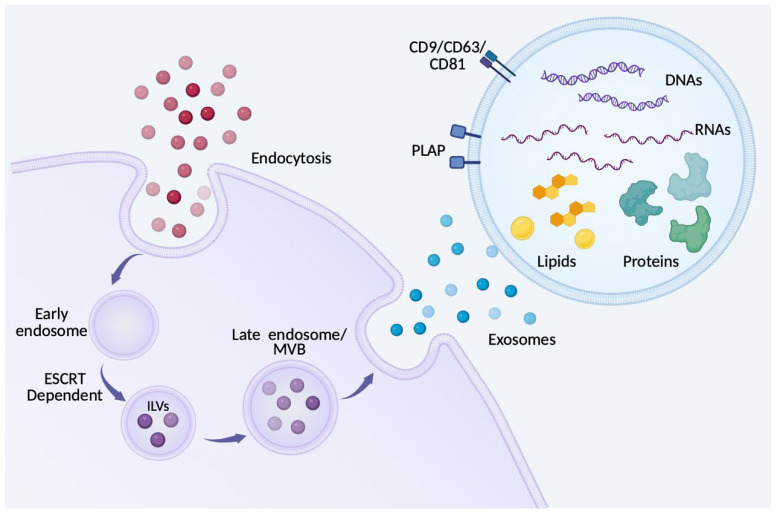
Biogenesis pathways and characteristic markers of placental exosomes, a major subtype of pdEVs. Exosomes originate from early endosomes formed by endosomal invagination, mature into multivesicular bodies (MVBs) during the late endosomal stage, and form intraluminal vesicles under the regulation of mechanisms such as the ESCRT complex. Following fusion of MVBs with the plasma membrane, intraluminal vesicles (ILVs) are released as exosomes. In addition to expressing universal exosome markers such as CD9, CD63, and CD81, placenta-derived exosomes specifically overexpress placental alkaline phosphatase (PLAP). Their contents include various bioactive molecules, including proteins and miRNAs. While this figure focuses on exosomes as the most extensively characterized EV subtype, the placenta also releases microvesicles and apoptotic bodies.

Exosomes, ranging from 40 to 160 nm in diameter, represent the smallest subtype of extracellular vesicle and are of endosomal origin, as shown in Figure 1 Their biogenesis involves a dual invagination process of the plasma membrane, leading to the formation of intracellular multivesicular bodies (MVBs) that encapsulate intraluminal vesicles (ILVs). These ILVs are ultimately released as exosomes via the fusion of MVBs with the plasma membrane and subsequent exocytosis [14,15]. Exosomal cargo—comprising DNA, RNA, lipids and proteins [16]—plays key roles in modulating immune responses, antigen presentation, and vascular homeostasis [35].

The most common method for exosome purification is high-speed ultracentrifugation, which does not fully discriminate between exosomes and other vesicular structures. Therefore, the detection of a specific biomarker is essential. Due to their endosomal origin, exosomes are enriched in CD63, CD9, and CD81, which can be detected by immunoelectron microscopy, flow cytometry, or Western blotting [36,37]. For pdEVs, placental alkaline phosphatase (PLAP) serves as a specific marker, enabling their isolation for further analysis [38]. However, methodological heterogeneity across studies—including differences in centrifugation protocols, storage conditions, and quantification methods—poses challenges for cross-study comparability. The International Society for Extracellular Vesicles (ISEV) has published Minimal Information for Studies of Extracellular Vesicles (MISEV) guidelines (2023) to address these standardization needs [39].

#### 2.3.2. PdEVs in Normal Pregnancy

Research has shown that the concentration of EVs in the circulation of pregnant women is more than 50-fold higher than that in non-pregnant women [40], suggesting a crucial role for EVs in maternal–fetal communication and fetal development. The placenta serves as a significant source of EVs during pregnancy [17]. Placental EVs are detectable in the maternal circulation as early as the first trimester of pregnancy, with their concentration progressively increasing until delivery. In the first trimester, placental exosomes transfer NKG2D receptor ligands, which suppress the expression of the NKG2D receptor on NK, CD8^+^, and γδT cells. This downregulation decreases receptor-mediated cytotoxicity and helps prevent maternal immune rejection of the fetus. Beyond this generalized immunosuppressive effect, placental EVs play a more nuanced role in immune tolerance through the presentation of non-classical MHC molecules.

A growing body of evidence indicates that placental EVs carry non-classical MHC class Ib molecules—specifically HLA-E, HLA-F, and HLA-G. These EV-associated HLA molecules can be transferred to maternal uterine natural killer (uNK) cells, where they engage inhibitory receptors such as NKG2A/CD94. This interaction suppresses uNK cell activation and cytotoxicity, thereby promoting immune tolerance toward the semi-allogeneic fetus. Importantly, this tolerance is not absolute; uNK cells retain the ability to recognize and eliminate microbial infections through activating receptors (e.g., NKG2D), highlighting a finely tuned balance between tolerance and defense. Disruption of this balance has been implicated in pregnancy complications, including recurrent miscarriage and preeclampsia [41,42].

PdEVs further contribute to the remodeling of endometrial spiral arteries during pregnancy, facilitating the exchange of gases and nutrients critical for fetal development. Moreover, pdEVs protect the fetus from viral infection through *C19MC* miRNA, which can be transported to recipient cells to manipulate viral resistance [43].

## 3. Maternal Nutritional Disturbances and pdEV Reprogramming

### 3.1. Nutritional Disturbances Alter pdEV Quantity and Composition

As discussed above, the early nutritional environment induces adaptive changes in the placenta. Further research revealed that these conditions also affect the concentration and biochemical composition of pdEVs, representing a key mechanism underlying placental adaptation.

GDM is a prevalent complication of pregnancy whose pathogenesis remains incompletely understood. Recent studies have indicated that pdEVs are critically involved in the initiation and progression of GDM. In GDM, the quantity, composition, and function of pdEVs significantly change [44]. Multiple studies have reported elevated concentrations of pdEVs in the plasma of GDM patients, along with notable alterations in the expression profiles of their cargo (e.g., miRNAs and proteins) [45,46]. The potential utility of pdEVs as early predictors of GDM has been highlighted in the literature. In a comprehensive review, Powe (2017) summarized that placenta-derived exosomes, along with other placental markers such as follistatin-like-3 and placental growth factor, represent promising candidates for first-trimester prediction of GDM [47]. These changes are hypothesized to be associated with placental stress responses under hyperglycemic conditions, although direct causal evidence is lacking.

**Figure 2:**
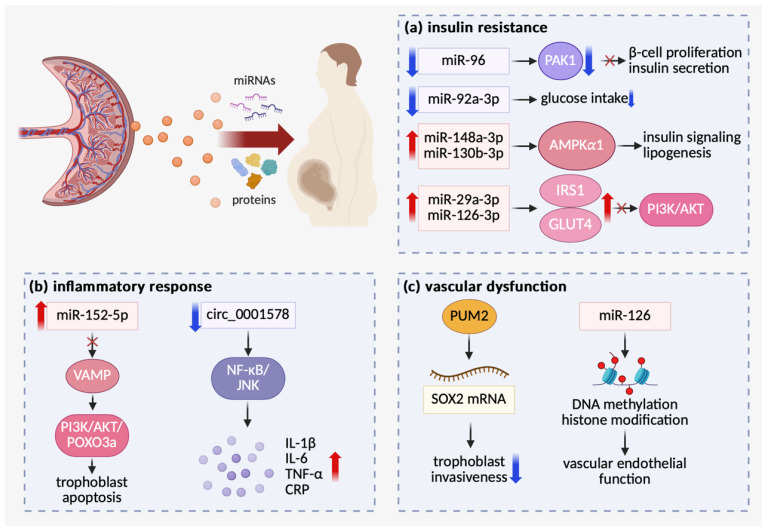
The mechanisms by which pdEVs mediate maternal metabolic dysregulation in GDM. This figure illustrates the multi-dimensional molecular networks through which pdEVs are hypothesized to influence maternal metabolic homeostasis in GDM. (**a**) Insulin Resistance Network: ① Downregulation of miR-96 attenuates its pro-proliferative effect on PAK1; ② miR-148a-3p/130b-3p disrupts insulin signaling via AMPKα1; ③ miR-29a-3p and others directly induce resistance via the IRS1-PI3K/AKT-GLUT4 axis; ④ miR-92a-3p downregulation produces a compensatory pro-glucose uptake effect. (**b**) Inflammatory Response Network: ① miR-152-5p promotes apoptosis and inflammation via the VAMP3-PI3K/AKT/FOXO3a axis; ② circ_0001578 downregulation activates the NF-κB/JNK pathway. (**c**) Vascular Dysfunction Network: Endothelial-derived exosomal PUM2 protein impairs trophoblast function by degrading SOX2, while inflammatory factor cascades synergistically cause vascular endothelial injury and epigenetic memory. ↑, upregulation/enrichment; ↓, downregulation/reduction.

In GDM, the miRNA expression profiles of pdEVs exhibit marked abnormalities. Various studies have reported differential expression of miRNAs, including miR-130b-3p, miR-320b, miR-135a-5p, miR-125b, miR-144, miR-140-3p, miR-574-3p, and miR-152-5p, as shown in Table 1. These miRNAs regulate target gene expression and influence multiple physiological processes, including angiogenesis, insulin secretion, apoptosis, and inflammatory responses, as shown in Figure 2. Once these “reprogrammed” EVs enter the maternal circulation, they can traverse biological barriers and be selectively taken up by specific target cells, potentially disseminating pathological signals from the placenta to multiple maternal systems. However, early studies in this field have been retracted due to data concerns [48], undergoing the need for more rigorous and standardized separation and quantification methods [49]. Furthermore, it is important to note that most reported miRNA changes represent clinical associations rather than established causal relationships.

Beyond GDM, altered pdEV profiles have also been reported in other pregnancy complications. In preeclampsia, pdEVs show increased levels of anti-angiogenic factors (e.g., sFlt-1, endoglin) and altered expression of miRNAs involved in endothelial function [50]. In preterm labor, pdEVs from women with intra-amniotic inflammation exhibit distinct proinflammatory cargo profiles [51]. These observations suggest that pdEV signatures may be complication-specific, though cross-condition comparisons remain limited.

**Table 1:** Differential expression profiles of placenta-derived extracellular vesicles across pregnancy complications.

References	Pregnancy Complication	Key Different Expressed Cargo	Main Biological Effects	Evidence Level
[52]	GDM	↑ miR-320b	β-cell dysfunction, insulin secretion inhibition, apoptosis promotion	Clinical association + in vitro
[53]	GDM	↑ miR-135a-5p	Activates PI3K/AKT via SIRT1, promotes trophoblast proliferation/invasion	In vitro
[54,55]	GDM	↑ miR-29a-3p, ↓ miR-92a-3p	Induce insulin resistance via IRS-1/PI3K/AKT/GLUT4 axis	In vitro + animal
[56,57]	GDM	↑ miR-152-5p, ↓ circ_0001578	Trophoblast apoptosis, NF-κB/JNK activation, proinflammatory cytokine release	In vitro
[58,59,60]	GDM	↑ LRG1, ECM1; ↑ miR-130b-3p; ↓ miR-140-3p, miR-574-3p	Bidirectional angiogenesis regulation (pro- and anti-angiogenic)	In vitro + animal
[61]	GDM	Altered miRNA profile (e.g., hsa-miR-149-3p, miR-455-3p)	Associated with insulin secretion, lipolysis, adipokine signaling	Clinical association
[62]	GDM	Specific miRNA signature (miR-125b, miR-144)	Diagnostic model with AUC 0.898 for GDM	Clinical association
[63,64]	GDM	Altered proteome (sirtuin signaling, oxidative phosphorylation)	Regulates placental glucose metabolism (glycolysis/gluconeogenesis)	Clinical association
[65]	GDM	↓ miR-516-5p, miR-517-3p, etc.	Non-invasive diagnostic potential in late pregnancy	Clinical association
[50]	PE	↑ sFlt-1, endoglin; altered angiogenic miRNAs	Anti-angiogenesis, endothelial dysfunction	Clinical association
[66,67,68,69]	PE	↑ NEP (neprilysin); ↑ miR-125b, miR-15a-5p; ↓ miR-31-5p, miR-199a-3p	Endothelial barrier injury, trophoblast dysfunction, hypertension	In vitro + animal
[70]	PE	↑ miR-372-3p	Induces mitochondrial damage in RVLM neurons	Animal
[69]	PE	↓ miR-199a-3p	Hinders VEGF-induced fetal glomerular dysplasia	Clinical association
[71]	PE	miR-520a-5p levels	Predicts severe PE (AUC = 0.806)	Clinical association
[72]	PE	Increased exosome concentration	Early biomarker before symptom onset (AUC 0.745–0.829)	Clinical association
[73]	PTL	Altered proteomic profile	Unique protein signatures in term vs. preterm labor	Clinical association
[74]	PTL	↓ sFLT-1, ↓ PlGF; ↑ sFLT-1/PlGF ratio	Imbalanced angiogenesis, potential predictive biomarker	Clinical association
[75]	PTL	Altered miRNA profile	Inhibited endothelial migration, promoted TNF-α release; mimics poor spiral artery remodeling	In vitro
[76]	PTL	↓ miR-519c (*C19MC* member)	Loss of endotoxin tolerance, uncontrolled inflammation leading to PTB	Clinical association + in vitro
[77]	Environmental pollutant exposure	↑ HMGB1, MAPK14 (alarmins)	Cellular injury signals, placental dysfunction, PTB risk	In vitro

Note: ↑, upregulation/enrichment; ↓, downregulation/reduction; AUC, area under the curve. GDM, gestational diabetes mellitus; PE, preeclampsia; PTL/PTB, preterm labor/preterm birth; *C19MC*, chromosome 19 microRNA cluster; NEP, neprilysin; sFLT-1, soluble fms-like tyrosine kinase-1; PlGF, placental growth factor; RVLM, rostral ventrolateral medulla; HMGB1, high mobility group box 1; MAPK14, mitogen-activated protein kinase 14; TNF-α, tumor necrosis factor-α.

### 3.2. PdEVs Influence Maternal Metabolism: Evidence and Knowledge Gaps

The most direct impact for pdEV effects on maternal metabolism comes from studies of pancreatic β-cells damage and insulin resistance. In GDM patients, multiple miRNAs are dysregulated in pdEVs: miR-320b is upregulated and its levels correlate positively with maternal blood glucose, suggesting a role in β-cell dysfunction; miR-96 is downregulated, potentially impairing β-cell proliferation via its target PAK1 [78]; miR-29a-3p is upregulated and associated with insulin resistance through the IRS-1/PI3K/AKT/GLUT4 axis; and miR-92a-3p is downregulated, potentially representing a compensatory adaptation that enhances glucose uptake capacity [54,55]. This contradictory phenomenon reflects the complexity and plasticity of metabolic regulatory networks.

Beyond miRNAs, proteomic studies have identified additional candidates: Jayabalan et al. reported that PAPP-A (pappalysin-1) is downregulated and CAMK2β (calcium/calmodulin-dependent protein kinase IIβ) is upregulated in GDM exosomes, both linked to insulin sensitivity [79].

Despite these findings, several important knowledge gaps remain. Most evidence derives from in vitro studies or clinical correlations without functional validation; the relative contribution of pdEVs versus other sources of circulating EVs is unclear; and direct evidence linking specific pdEV cargo to maternal metabolic outcomes in longitudinal cohorts is limited. For detailed mechanistic pathways—including inflammation, angiogenesis, and epigenetic effects—the reader is referred to the integrated functional domains presented in Section 4.

## 4. Functional Mechanisms of pdEVs in Metabolic Programming

Building upon the alterations in pdEV quantity and composition described in Section 3, this section synthesizes the current evidence on how these reprogrammed EVs exert their biological effects on both maternal and fetal systems. We will discuss possible mechanisms in metabolic programming by functional domain. Four core domains are discussed in sequence: metabolic reprogramming (insulin sensitivity and β-cell function), lipid metabolism (the PPARγ axis), angiogenesis and vascular remodeling, and inflammatory and immune regulation. A fifth subsection addresses the emerging—but still hypothetical—role of pdEVs in epigenetic programming. Within each functional domain, evidence from maternal and offspring studies is integrated to highlight how pdEVs may simultaneously or sequentially influence metabolic health across generations. Table 2 provides a comprehensive summary of the key pdEV cargo molecules discussed in this section, their proposed targets and mechanisms, and the evidence levels supporting each finding.

### 4.1. Metabolic Reprogramming: Insulin Sensitivity and β-Cell Function

The PI3K/Akt signaling pathway serves as a central hub integrating insulin signaling, cell proliferation, and metabolic regulation. PdEVs from GDM patients have been shown to modulate this pathway in both maternal and fetal pancreatic tissues, albeit with distinct functional consequences. In the maternal compartment, as introduced in Section 3.2, upregulated miR-320b, downregulated miR-96, upregulated miR-29a-3p, and downregulated miR-92a-3p collectively impair insulin secretion and signaling, contributing to the systemic insulin resistance that characterizes GDM. These effects are mediated primarily through the IRS-1/PI3K/Akt/GLUT4 axis, with miR-29a-3p directly targeting IRS-1 and GLUT4, while miR-92a-3p downregulation may represent a compensatory adaptation.

In the fetal compartment, evidence from mouse models suggests that pdEVs can enter the fetal pancreas and influence islet cell development through similar PI3K/Akt pathway modulation but with different developmental consequences [88]. A specific miRNA, designated miR-7-19488, in placental exosomes is upregulated in pdEVs from GDM patients. Based primarily on mouse studies, this miRNA targets *PIK3R2* mRNA, reducing p85β protein expression and thereby decreasing PI3K activity [80]. The resulting persistent activation of the downstream PI3K-Akt-FoxO1/mTORC1 signaling pathway has been associated with both increased β-cell proliferation and premature functional maturation before birth [89,90,91,92].

Concurrently, GDM-derived exosomes increase glucagon-like peptide-1 (GLP-1) synthesis in fetal pancreatic α-cells, activating the local GLP-1/GLP-1R axis and further enhancing PI3K-Akt signaling through G protein-coupled mechanisms [90,93,94].

Thus, while maternal pdEVs contribute to insulin resistance (a maladaptive response), fetal pdEVs may drive excessive islet development and premature maturation (a programming event with potential long-term consequences). Several caveats must be emphasized: most fetal evidence derives from mouse models, human fetal data are extremely limited, and direct evidence that pdEVs—rather than other placental factors—mediate these effects in humans is lacking. The long-term consequences of premature β-cell maturation for adult metabolic health remain hypothetical, and the relationship between maternal insulin resistance and fetal β-cell programming via shared PI3K/Akt pathway modulation requires further investigation in human cohorts.

### 4.2. Lipid Metabolism: The PPARγ Axis

Peroxisome proliferator-activated receptor gamma (PPARγ) is a master regulator of fatty acid storage, glucose metabolism, and insulin sensitivity that has emerged as a key mediator through which pdEVs may regulate metabolic programming, with effects predominantly on the fetus rather than the mother [87]. Dysregulation of PPARγ has been associated with GDM, and elevated PPARγ gene expression has been reported in placentas from GDM patients [95,96]. Observational data indicate that human placental PPARγ expression is positively correlated with placental weight and fetal weight [97]. Beyond nutrient sensing, PPARγ participates in trophoblast differentiation, HCG secretion, and regulation of nutrient transport from placenta to fetus [98,99,100]. Recent findings from mouse models provide mechanistic insights into how pdEV-associated PPARγ may influence fetal metabolism. Luo and colleagues demonstrated that PPARγ protein carried by pdEVs can enter the nuclei of fetal preadipocytes, where it activates downstream adipogenesis-related genes (e.g., *Arid5b*, *Rorb*, *Pank2*, and *Htr2c*) [87]. This transcriptional activation promotes adipocyte differentiation and fat deposition, thereby influencing fetal subcutaneous fat mass and birth weight. Additionally, PPARγ activation in human trophoblasts increases free fatty acid uptake and upregulates fatty acid transport proteins, facilitating lipid transfer from the placenta to the fetus [101]. The same study revealed that the PPARγ agonist rosiglitazone (RGZ) or antagonist GW9662 can modulate trophoblast differentiation in vitro, suggesting a potential pharmacological avenue for regulating intrauterine fat formation. Notably, direct evidence for PPARγ-mediated effects on maternal lipid metabolism via pdEVs is limited, suggesting that the primary impact of this pathway is on fetal programming. Important caveats include the lack of direct evidence that PPARγ transfer via pdEVs occurs in human pregnancy, the need for validation of nuclear PPARγ transfer in human tissues, and the unknown safety of PPARγ-modulating agents during pregnancy. Therefore, while the PPARγ axis represents a promising area for future research, clinical translation would require extensive safety evaluation and human validation.

### 4.3. Angiogenesis and Vascular Remodeling

PdEVs from GDM patients exhibit apparently contradictory yet potentially coordinated effects on the vascular system. This bidirectional regulation operates in both maternal and placental vascular beds, with distinct functional consequences at each site. In the maternal vascular compartment, the hyperglycemic environment of GDM induces endothelial cells to release EVs carrying the PUM2 protein, which reduces trophoblast invasiveness and impairs spiral artery remodeling, increasing the risk of GDM complicated by preeclampsia [86]. Additionally, EV-delivered miR-126 may contribute to maternal “metabolic memory” through epigenetic effects on vascular endothelial function genes, though this remains a hypothesis [84,85].

In the placental vascular compartment, which directly impacts fetal nutrient supply, GDM-derived pdEVs carry both anti-angiogenic miRNAs and pro-angiogenic proteins that act on the placental vasculature. On the anti-angiogenic side, miR-130b-3p inhibits endothelial cell adhesion and migration by targeting ICAM-1 [59], while miR-140-3p and miR-574-3p suppress angiogenesis signaling by targeting VEGF and its receptors [60]. On the pro-angiogenic side, leucine-rich alpha-2-glycoprotein-1 (LRG1) activates the non-canonical TGF-β/Smad1/5/8 pathway, upregulating VEGFR2 and angiopoietin-2 to drive excessive proliferation and abnormal branching of placental capillaries [58,102,103,104,105]. Extracellular matrix protein 1 (ECM1) also promotes angiogenesis, though its specific mechanisms remain incompletely elucidated. The functional consequence of these opposing signals is a high-resistance, low-efficiency vascular network that, despite vessel density, may ultimately lead to reduced placental perfusion efficiency. Thus, while maternal vascular effects of pdEVs primarily involve spiral artery remodeling and endothelial dysfunction, the placental vascular effects directly impact fetal nutrient delivery and growth. The net balance between anti- and pro-angiogenic signals in human GDM remains unclear, and direct evidence linking altered placental angiogenesis to offspring metabolic outcomes is lacking.

### 4.4. Inflammatory and Immune Regulation

Inflammation represents a common mechanistic thread linking maternal metabolic dysfunction and fetal programming, with pdEVs serving as key vectors for transmitting inflammatory signals across the placenta [106].

In the porcine model of early pregnancy, the conceptus (embryo and associated membranes) packages interferon gamma (IFNG) into EVs. These IFNG-containing EVs cross the luminal epithelium and enter the underlying stroma, where IFNG promotes recruitment of T cells to the endometrium [107]. This controlled inflammatory response is required for successful implantation in this species. It supports the hypothesis that EV-mediated signaling is a fundamental mechanism for establishing controlled inflammation at the maternal–fetal interface.

In the maternal compartment, upregulated miR-152-5p in GDM pdEVs suppresses VAMP3 protein expression, inhibiting the PI3K/AKT/FOXO3a pathway and increasing trophoblast apoptosis [108]. This may lead to placental dysfunction and local inflammation. Downregulation of circ_0001578 relieves normal inhibition of NF-κB and JNK signaling, inducing the synthesis and release of proinflammatory cytokines, including IL-1β, IL-6, TNF-α, and CRP, which can directly impair maternal insulin signaling [56,57,109]. These inflammatory factors may further stimulate the placenta to produce more proinflammatory EVs, potentially creating a self-amplifying inflammatory cycle [110,111]. The adipokine chemerin, elevated in GDM, downregulated miR-140-3p and miR-574-3p in EVs, releasing their normal inhibition of VEGF and establishing crosstalk between angiogenesis and inflammation [60]. Additionally, altered pdEV signaling promotes monocyte chemotaxis to the placenta and maternal adipose tissue, driving polarization toward the proinflammatory M1 phenotype. These activated macrophages release inflammatory mediators and propagate inflammatory signals via their own EVs, amplifying the inflammatory response [112]. In normal pregnancy, pdEVs deliver immunoregulatory miRNAs (e.g., miR-146a, miR-155) to promote maternal T-cell differentiation and maintain immune tolerance [113]. In GDM, the altered expression of these regulatory miRNAs enriches proinflammatory components. Moreover, the expression of HLA molecules on pdEVs may be altered, potentially compromising uNK-mediated tolerance, though direct evidence is lacking. This disruption may impair placental integrity and transmit inflammatory signals to the fetus [61].

Regarding fetal effects, studies in obese mouse models have shown that pdEVs attenuate endoplasmic reticulum stress markers (e.g., GRP78, p-eIF2α, and CHOP) in fetal tissues while inhibiting NF-κB and HIF-1α activation and reducing TNF-α and IL-6 levels [83,114]. Specific miRNAs implicated in these fetal effects include miR-499 (which inhibits the Lin28B/let-7/NF-κB axis [82]); let-7i-5p (which suppresses NF-κB and mitochondrial dysfunction) [83]; and miR-15b-5p (which suppresses Apelin and upregulates proinflammatory cytokines) [81]. These miRNAs may be delivered to fetal tissues via pdEVs, potentially influencing the establishment of insulin signaling pathways and glucose homeostasis during fetal development [115]. The same pdEVs that drive maternal inflammation may also directly expose the fetus to inflammatory signals, creating a continuum of metabolic risk from mother to offspring. However, direct evidence that pdEVs transmit maternal inflammatory states to human offspring is lacking, human fetal tissue data are extremely limited, and the causal link between pdEV-mediated inflammation and offspring glucose metabolism requires validation in human cohorts.

### 4.5. Epigenetic Programming: Hypotheses and Emerging Evidence

**Figure 3:**
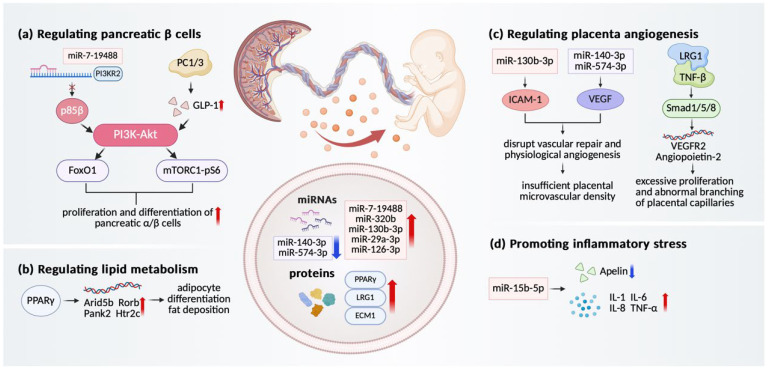
Key molecular mechanisms by which pdEVs are proposed to regulate fetal metabolic programming. Based primarily on evidence from mouse models and in vitro studies, this figure illustrates four core pathways through which pdEVs may influence fetal metabolic programming under adverse early nutritional conditions (e.g., GDM). (**a**) Pancreatic β-cell pathway (predominantly from mouse studies): Upregulated miR-7-19488 in exosomes targets and inhibits *PIK3R2*, releasing suppression of the PI3K-Akt-FoxO1/mTORC1 pathway. This has been associated with excessive β-cell proliferation and premature functional maturation. (**b**) Lipid metabolism pathway (mouse model): EV-delivered PPARγ protein enters the nuclei of fetal preadipocytes, activating adipogenesis genes such as *Arid5b* and *Rorb* to promote lipid deposition. (**c**) Angiogenesis pathway (in vitro and animal evidence): Bidirectional regulatory mechanisms include ① miR-130b-3p, miR-140-3p, and miR-574-3p, which suppress normal vascular repair by targeting ICAM-1, VEGF, and others; ② pro-angiogenic proteins like LRG1 drive abnormal capillary proliferation. (**d**) Inflammatory stress pathway (in vitro evidence): Exosomal miR-15b-5p suppresses Apelin signaling, thereby upregulating proinflammatory factors such as IL-1, IL-6, and TNF-α. ↑, upregulation/enrichment; ↓, downregulation/reduction.

As previously described, pdEVs may regulate glucose metabolism programming in offspring through multiple pathways, as shown in Figure 3. The hypothesis that pdEVs establish enduring molecular memory in fetal tissues through epigenetic mechanisms bridges maternal and offspring effects across generations. This concept, often termed “metabolic memory,” posits that non-coding RNAs—particularly miRNAs—delivered via pdEVs persistently remodel fetal metabolic tissue gene expression profiles, potentially increasing offspring susceptibility to insulin resistance and T2DM later in life.

In GDM models, pdEVs exhibit characteristic alterations in miRNA expression profiles, including significant upregulation of miR-148a-3p, miR-130b-3p, miR-29a-3p, and miR-126-3p [84]. The proposed mechanism, based largely on in vitro studies and inference from other biological systems, is that pdEVs deliver regulatory molecules to differentiating fetal tissues during specific developmental windows of high epigenetic plasticity [116]. Unlike transient signals such as hormones, miRNAs delivered by pdEVs integrate into the RNA-induced silencing complex (RISC) of fetal cells. They may persist across multiple cell divisions through incomplete dilution during mitosis. As discussed in Section 4.2, pdEV-delivered PPARγ protein can enter fetal preadipocyte nuclei and activate adipogenesis-related genes, representing a non-canonical epigenetic mechanism that may also contribute to persistent metabolic programming.

However, a hierarchy of evidence is essential when interpreting epigenetic programming claims. Currently, no mechanism in the pdEV field meets the standard of “established” (replicated human studies with functional validation). The PPARγ transfer findings in mice and the miRNA expression differences observed in human pdEVs represent “emerging” evidence—consistent animal data with some human correlational support. The concept of “metabolic memory” persisting across cell divisions remains at the hypothetical level, lacking direct experimental validation in the context of pdEV biology [9]. Direct evidence from human longitudinal studies—tracking pdEV exposure in utero and metabolic outcomes decades later—is currently lacking. Therefore, while the epigenetic programming hypothesis provides an attractive framework for understanding how maternal metabolic disturbances influence offspring health across generations, it should be viewed as a guide for future investigation rather than an established mechanism.

## 5. Summary, Knowledge Gap, and Future Directions

### 5.1. Summary of Key Findings

**Figure 4:**
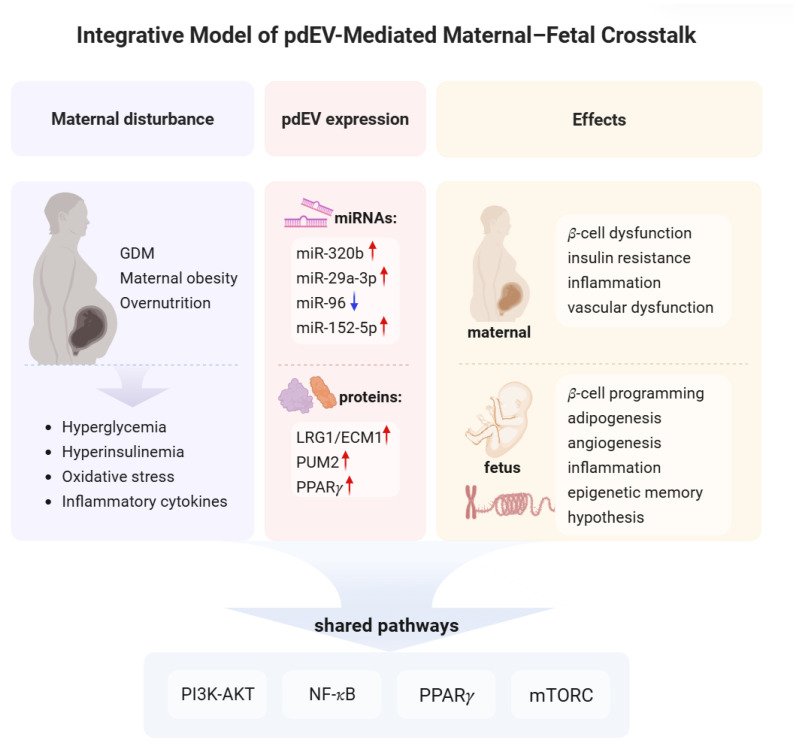
Integrative model of pdEV-mediated maternal–fetal crosstalk. Under adverse early nutritional conditions such as GDM, maternal obesity, and overnutrition, placental cells respond to hyperglycemia, hyperinsulinemia, oxidative stress, and inflammatory cytokines by reprogramming the cargo of secreted pdEVs. These pdEVs carry altered expression profiles of miRNAs (e.g., upregulated miR-320b, miR-29a-3p, miR-152-5p; downregulated miR-96) and proteins (e.g., upregulated LRG1, ECM1, PUM2, PPARγ). Upon reaching target cells, pdEVs exert effects on both maternal and fetal systems. Maternal effects include pancreatic β-cell dysfunction, insulin resistance, inflammatory activation, and vascular dysfunction. Fetal effects include premature β-cell maturation, increased adipogenesis, abnormal placental angiogenesis, and inflammatory programming. Meanwhile, the epigenetic memory hypothesis may contribute to fetal metabolic programming. Shared signaling pathways including PI3K-AKT, NF-κB, PPARγ, and mTORC1 mediate these effects across both compartments. ↑, upregulation/enrichment; ↓, downregulation/reduction.

The early stages of life represent a critical period for human growth and development, during which the fetus is highly susceptible to adverse environmental influences. The placenta serves not merely as a passive conduit but as an active sensor and processor of maternal nutritional signals. The evidence synthesized in this review suggests that placenta-derived extracellular vesicles (pdEVs) respond to maternal nutritional disturbances—such as GDM and obesity—by altering their cargo composition, and these changes may mediate effects on offspring metabolic programming. Figure 4 summarizes the integrative model of pdEV-mediated maternal fetal crosstalk. The putative mechanisms identified in the literature include regulation of pancreatic β-cell development (primarily from mouse models), modulation of lipid metabolism via the PPARγ axis (promising animal data), bidirectional regulation of placental angiogenesis (in vitro evidence with unclear net balance), promotion of inflammatory and oxidative stress responses (multiple miRNA mediators identified), and epigenetic programming hypotheses (theoretically plausible but lacking human validation). Based on these observations, pdEV profiles have been proposed as potential early biomarkers for predicting metabolic risk in offspring. However, as detailed below, substantial knowledge gaps remain before clinical translation can be realistically considered.

### 5.2. Major Knowledge Gaps

Several major knowledge gaps must be acknowledged. First, direct evidence linking pdEVs to offspring metabolic outcomes in humans is extremely limited, as most current evidence derives from maternal circulation studies or in vitro experiments, and the extrapolation from mouse models to human physiology requires caution. Second, the majority of reported miRNA and protein changes represent clinical associations rather than established causal relationships; functional validation using genetic knockout or inhibition studies in appropriate models is lacking for most candidate cargo molecules. Third, methodological heterogeneity across studies—including differences in EV isolation protocols (ultracentrifugation vs. size-exclusion chromatography), storage conditions, quantification methods, and normalization strategies—poses challenges for cross-study comparability, despite the availability of MISEV guidelines. Fourth, no studies have tracked pdEV exposure in utero and subsequent metabolic outcomes in offspring beyond early childhood, leaving the hypothesized “metabolic memory” untested in humans. Fifth, most studies pool all small EVs under the term “exosomes” without distinguishing functional differences between exosomes, microvesicles, and other EV populations. Sixth, maternal nutritional conditions beyond GDM—including obesity without GDM, undernutrition, and specific dietary component effects—remain poorly characterized.

### 5.3. Future Research Directions

Based on these knowledge gaps, we propose the following prioritized research directions. The highest priority is prospective cohort studies with offspring follow-up: longitudinal cohorts collecting maternal blood during pregnancy and following offspring metabolic outcomes into adolescence and adulthood are needed to establish whether pdEV profiles in pregnancy predict offspring metabolic health. Second, causal validation in animal models—using genetic knockout or inhibition of specific miRNAs/proteins in pdEVs, as well as adoptive transfer experiments—is required to determine whether specific cargo molecules are necessary and sufficient for programming effects. Third, methodological standardization following MISEV 2023 guidelines [39] would enable cross-study meta-analyses and validation. Fourth, single-EV and subtype-specific analyses using technologies such as nano-flow cytometry and single-EV sequencing could distinguish the functional roles of exosomes versus microvesicles in maternal–fetal communication. Fifth, comparative studies across nutritional perturbations—head-to-head comparisons of GDM, obesity, undernutrition, and specific dietary exposures—could identify common versus condition-specific pdEV signatures. Sixth, mechanistic studies on epigenetic programming, including longitudinal sampling of offspring tissues in animal models and assessment of DNA methylation, histone modifications, and non-coding RNA persistence, are needed to rigorously test the “metabolic memory” hypothesis.

### 5.4. Clinical Translation Prospects: Opportunities and Limitations

Regarding clinical translation, several limitations must be acknowledged. No pdEV-based test is near clinical use, therapeutic targeting during pregnancy raises safety concerns, and the lack of standardized methods poses major barriers. A realistic near-term outlook includes refinement of isolation methods, establishment of normative pdEV ranges across healthy pregnancy, and validation of candidate biomarkers in multi-center cohorts.

In summary, pdEVs have emerged as intriguing intermediaries in maternal–fetal communication that may influence offspring metabolic programming. However, the current evidence base remains largely descriptive and associative, with substantial knowledge gaps in causal validation, human translation, and long-term outcomes. Leveraging the maternal–fetal communication role of pdEVs holds theoretical promise for improving metabolic health across generations, but realizing this potential will require sustained investment in foundational research, method development, and longitudinal human cohorts. For now, the field would benefit most from cautious interpretation of existing data, replication of key findings, and adherence to evidence-level transparency.

## 6. Conclusions

Placental extracellular vesicles (pdEVs) mediate maternal–fetal communication and may influence offspring metabolic programming under adverse early nutrition. Current evidence from animal models and clinical associations suggests pdEVs modulate β-cell development, lipid metabolism, angiogenesis, and inflammation. However, major knowledge gaps remain in causal validation and human translation. Prospective cohort studies and standardized methodologies are urgently needed.

## Figures and Tables

**Table 2:** Key cargo of placenta-derived extracellular vesicles and their metabolic effects with evidence levels.

References	Cargo & Direction	Target/Mechanism	Maternal Effect	Offspring Effect	Experimental Validation Level
miRNAs					
[52]	miR-320b ↑	Unknown	β-cell dysfunction, inhibits insulin secretion, promotes apoptosis	Not studied	Inferred from expression data + clinical association
[78]	miR-96 ↓	PAK1	Impaired β-cell proliferation, reduced insulin secretion	Not studied	Experimentally validated (in vitro)
[55]	miR-29a-3p ↑	IRS-1, GLUT4, PPARδ	Insulin resistance via IRS-1/PI3K/AKT/GLUT4 axis	Not studied	Experimentally validated (in vitro + animal)
[54]	miR-92a-3p ↓	Insulin signaling pathway	Enhanced glucose uptake capacity (compensatory adaptation)	Not studied	Inferred from expression data
[56]	miR-152-5p ↑	VAMP3 → PI3K/AKT/FOXO3a	Trophoblast apoptosis, placental inflammation	Not studied	Experimentally validated (in vitro)
[59]	miR-130b-3p ↑	ICAM-1	Impaired endothelial adhesion/migration	Reduced placental angiogenesis	Experimentally validated (in vitro)
[60]	miR-140-3p ↓	VEGF	Reduced pro-angiogenic signaling	Reduced placental angiogenesis	Experimentally validated (in vitro)
[60]	miR-574-3p ↓	VEGF	Reduced pro-angiogenic signaling	Reduced placental angiogenesis	Experimentally validated (in vitro)
[80]	miR-7-19488 ↑	PIK3R2 → PI3K/Akt/mTORC1	Not studied	Premature β-cell maturation, excessive proliferation	Experimentally validated (in vitro mouse)
[81]	miR-15b-5p ↑	Apelin signaling pathway	Proinflammatory cytokine upregulation (IL-1, IL-6, TNF-α)	Not studied	Experimentally validated (in vitro)
[82]	miR-499 ↑	Lin28B/let-7/NF-κB axis	Anti-inflammatory, anti-oxidative stress	Not studied	Experimentally validated (in vitro)
[83]	let-7i-5p ↑	NF-κB, mitochondrial function	Anti-inflammatory, anti-apoptotic	Attenuates ER stress in fetal tissues	Experimentally validated (in vitro mouse)
[84,85]	miR-126 ↑/↓ (dysregulated)	DNA methylation, histone modification	Induces persistent vascular cell alterations (“metabolic memory”)	Not studied	Hypothesis
Circular RNAs					
[57]	circ_0001578 ↓	NF-κB, JNK pathways	Proinflammatory cytokine release (IL-1β, IL-6, TNF-α, CRP)	Not studied	Experimentally validated (in vitro)
Proteins					
[58]	LRG1 ↑	TGF-β/Smad1/5/8 pathway	Increased angiogenesis	Excessive placental vascularization	Experimentally validated (in vitro + animal)
[58]	ECM1 ↑	Unknown (angiogenesis-related)	Increased angiogenesis	Excessive placental vascularization	Experimentally validated (in vitro + animal)
[86]	PUM2 ↑	SOX2 mRNA degradation	Reduced trophoblast invasiveness, impaired spiral artery remodeling	Not studied	Experimentally validated (in vitro)
[87]	PPARγ ↑	*Arid5b*, *Rorb*, *Pank2*, *Htr2c* (adipogenesis genes)	Not studied	Increased adipogenesis, lipid deposition, fetal fat mass	Experimentally validated (in vitro mouse)
Proteomic findings					
[79]	PAPP-A ↓	Insulin sensitivity regulation	Associated with insulin resistance	Not studied	Inferred from expression data + clinical association
[79]	CAMK2β ↑	Calcium signaling, insulin sensitivity	Associated with decreased insulin sensitivity	Not studied	Inferred from expression data + clinical association

Note: ↑, upregulation/enrichment; ↓, downregulation/reduction. ER, endoplasmic reticulum; IL, interleukin; TNF-α, tumor necrosis factor-alpha; CRP, C-reactive protein; ICAM-1, intercellular adhesion molecule-1; VEGF, vascular endothelial growth factor; IRS-1, insulin receptor substrate 1; GLUT4, glucose transporter type 4; PAK1, p21-activated kinase 1; VAMP3, vesicle-associated membrane protein 3; SOX2, SRY-box transcription factor 2; LRG1, leucine-rich alpha-2-glycoprotein-1; ECM1, extracellular matrix protein 1; PPARγ, peroxisome proliferator-activated receptor gamma; PAPP-A, pappalysin-1; CAMK2β, calcium/calmodulin-dependent protein kinase IIβ. Experimental validation level definitions: clinical association = human correlational study (non-causal); in vitro = cell-based experiment; in vivo (mouse) = mouse model validation; in vitro + animal = both cell and animal evidence; hypothesis = proposed but not experimentally validated.

## Data Availability

No new data were created or analyzed in this study. Data sharing is not applicable to this article.
